# Performance of mitochondrial DNA mutations detecting early stage cancer

**DOI:** 10.1186/1471-2407-8-285

**Published:** 2008-10-03

**Authors:** John P Jakupciak, Samantha Maragh, Maura E Markowitz, Alissa K Greenberg, Mohammad O Hoque, Anirban Maitra, Peter E Barker, Paul D Wagner, William N Rom, Sudhir Srivastava, David Sidransky, Catherine D O'Connell

**Affiliations:** 1 Biochemical Science Division, National Institute of Standards and Technology, Gaithersburg, Maryland 20899, USA; 2 Geo-Centers, Inc, Newton, Massachusetts, USA; 3 Johns Hopkins University School of Medicine, 720 Rutland Ave, Baltimore, Maryland 21205, USA; 4 Cancer Biomarkers Research Group, National Cancer Institute, Rockville, Maryland, USA; 5 Division of Pulmonary and Critical Care Medicine, NYU, School of Medicine, New York, USA; 6 Tetracore, Inc, Rockville, Maryland, USA

## Abstract

**Background:**

Mutations in the mitochondrial genome (mtgenome) have been associated with cancer and many other disorders. These mutations can be point mutations or deletions, or admixtures (heteroplasmy). The detection of mtDNA mutations in body fluids using resequencing microarrays, which are more sensitive than other sequencing methods, could provide a strategy to measure mutation loads in remote anatomical sites.

**Methods:**

We determined the mtDNA mutation load in the entire mitochondrial genome of 26 individuals with different early stage cancers (lung, bladder, kidney) and 12 heavy smokers without cancer. MtDNA was sequenced from three matched specimens (blood, tumor and body fluid) from each cancer patient and two matched specimens (blood and sputum) from smokers without cancer. The inherited wildtype sequence in the blood was compared to the sequences present in the tumor and body fluid, detected using the Affymetrix Genechip^® ^Human Mitochondrial Resequencing Array 1.0 and supplemented by capillary sequencing for noncoding region.

**Results:**

Using this high-throughput method, 75% of the tumors were found to contain mtDNA mutations, higher than in our previous studies, and 36% of the body fluids from these cancer patients contained mtDNA mutations. Most of the mutations detected were heteroplasmic. A statistically significantly higher heteroplasmy rate occurred in tumor specimens when compared to both body fluid of cancer patients and sputum of controls, and in patient blood compared to blood of controls. Only 2 of the 12 sputum specimens from heavy smokers without cancer (17%) contained mtDNA mutations. Although patient mutations were spread throughout the mtDNA genome in the lung, bladder and kidney series, a statistically significant elevation of tRNA and ND complex mutations was detected in tumors.

**Conclusion:**

Our findings indicate comprehensive mtDNA resequencing can be a high-throughput tool for detecting mutations in clinical samples with potential applications for cancer detection, but it is unclear the biological relevance of these detected mitochondrial mutations. Whether the detection of tumor-specific mtDNA mutations in body fluidsy this method will be useful for diagnosis and monitoring applications requires further investigation.

## Background

The mitochondrial genome is well characterized and is composed of 16,568 base pairs, harboring 37 densely packed genes [1]. The mitochondrial genome has an accelerated mutation rate in comparison to the nuclear genome [2], and DNA repair is less efficient in the mitochondria than in the nuclear genome [3]. This high frequency of mutation and the high copy number of mitochondria per cell suggest that mtDNA mutations may be useful markers for personalized assessment of cancer. Since mitochondrial DNA lacks introns, mutations are likely to accumulate in the coding regions and have biological consequences [4].

Mutations in mitochondrial DNA (mtDNA) have been detected in colorectal, breast, cervical, ovarian, prostate, liver, pancreatic, and lung cancers [5-22]. Recently, the utility of a mtDNA deletion for molecular definition of benign, malignant and proximal to malignant prostate needle biopsies was reported [23]. These mitochondrial mutations may be linked to a field effect [24], which suggests that mutations in the mitochondrial genome may represent sensitive markers for neoplastic transformation [25]. In addition to cancer, mtDNA instabilities have been reported in degenerative diseases [26-29], neurodegenerative diseases [30,31], macular degeneration [32], aging and longevity [28,33-36] and cardiovascular disease [37].

Body fluids such as urine, which can be obtained by noninvasive techniques, are potential sources of biomarkers that are complementary to traditional sources such as serum or plasma [38]. Recent research demonstrated the feasibility of whole mtgenome analysis using nipple aspirate fluid (NAF) for breast cancer detection [39]. MtDNA mutations in the D-loop were also detected in urine sediments from bladder cancer patients [40]. In some cases, urine analysis for DNA mutations is more sensitive than plasma analysis [41].

Using fluorescence-based capillary electrophoresis (CE) sequencing of the full mitochondrial genome and rigorous quality control procedures [42], we confirmed mtDNA mutations in tumors of 5 of 11 (45%) lung cancer patients [7]. However, traditional automated sequencing is too laborious and lacks adequate resolution to detect mtDNA heteroplasmy that may be required to detect mtDNA from cancerous cells in the presence of normal cells [43,44]. The Affymetrix Genechip^® ^Human Mitochondrial Resequencing Array 1.0 (MitoChip) is faster, less expensive and allows for better resolution of mixtures than CE DNA sequencing. This microarray design provides replicate information within a single chip for the mtDNA coding region, as both forward and reverse strands are tiled in duplicate, resulting in a four-fold coverage for each nucleotide position of the mitochondrial genome. In this study, we compare the mtDNA from both tumor and associated body fluid with respect to that isolated from the patient's blood. mtDNA isolated from blood provides the normal control or germ-line sequence. To verify these results, CE DNA sequencing was performed on the three matched specimens from a subset of four patients.

The majority of previous studies reported tumor mtDNA mutations that were homoplasmic [45]. In contrast, the heightened sensitivity of the MitoChip allows for the detection of heteroplasmies (the condition where mutations coexist with wild-type genomes). Several recent publications have estimated that more than half of mtDNA sequence publications contain errors [46-48], some of which result from mismatched specimens. Consequently, we used STR analysis to ensure that the samples were accurately matched

Most previous studies have examined tumor tissue for mtDNA mutations, we extended this to evaluate the feasibility of using body fluids in proximity to the tumor as a non-invasive method to detect mtDNA mutations. Our results also suggest that mtDNA mutations in body fluids can partially distinguish lung cancer patients from heavy smokers.

## Methods

### Study Subjects

Specimens were collected from 26 cancer patients from Johns Hopkins University Hospital after informed consent and IRB approval; and 24 non-cancer smoker controls provided blood and sputum from New York University after informed consent and IRB approval. All DNA samples were prepared from cryostat sectioned frozen cancer tissues, which had been freed of non-neoplastic cells by microdissection. Portions of the primary tumor tissue were routinely embedded in optimum cold temperature medium and the frozen specimen block was then cut with a cryostat. Initially, two 5 um sections were obtained for hematoxilyn staining and then examined by light microscopy. Sections from the tumor were reviewed carefully and the neoplastic cell content recorded as a percentage of the total cells on a section. Next several 10 um sections are cut and placed in TE9 containing 1% SDS/Protenase K overnight. Following overnight incubation, DNA was extracted and precipitated as described previously [49]. Primary tumor samples containing less than 50% neoplastic cells were further microdissected to enhance the neoplastic cell content. 80% ethanol was placed over the lightly stained tissue on the slide and non-neoplastic cell containing regions was dissected away from the tumor cells with fine tissue forceps or a 30-gauge needle using an inverted microscope. Free floating cells are then washed off the slide with 80% alcohol and the remaining regions of neoplastic cells scraped off and processed in buffer. Extracted DNA was stored at -20°C. DNA from primary tumors, body fluids and blood was evaluated. For the collection of the sputum, the study subjects inhale normal saline mist and then cough up a specimen. For the collection of the sputum, the study subjects inhale normal saline mist and then cough up a specimen. The sputum is from the airways and contains alveolar macrophages and bronchial epithelial cells. The sputum is processed for protein analysis and cells are separated by centrifugation and then placed in TRIzol^®^. Bronchoalveolar lavage (BAL) is a saline wash introduced into a wedged bronchoscope and BAL cells retrieved in the saline. The cells are spun down and the BAL fluid stored (it contains 1% of epithelial lining fluid). The BAL cells are kept in TRIzol^®^. Both the sputum and BAL specimens contain alveolar macrophages.

### DNA Isolation

DNA from tumor sections was digested with 1% SDS/Proteinase K, extracted by phenol, chloroform and ethanol precipitated. Tumor samples were obtained from males and females. Samples were collected primarily from early stage (stage IA to IIB) tumors. We received DNA from 3 matched specimen samples from cancer patients. From 9 lung cancer patients, we received DNA from tumor, blood, and BALs (washings). From 4 bladder cancer and 14 kidney cancer patients, we received DNA from tumor, blood, and urine. Diagnosis and staging of patient tumors is provided in Table 1. In one patient sample set, DNA could not be amplified from the blood and the patient set was removed from analysis

**Table 1:** Cancer cohort: characteristics and number of mtDNA mutations identified

Sample	Age	Diagnosis	Stage	Blood/Tumor	Blood/Body Fluid*
Lung 1	54	SCC	IA	6	1
Lung 2	51	Adenocarcinoma	IA	3	n/a
Lung 3	72	Adenocarcinoma	IA	2 (D-Loop)	0
Lung 4	70	BALV CA	IA	2	0
Lung 5	77	SCC	IA	3	0
Lung 6	61	Adenocarcinoma	IIA	0	1
Lung 7	73	NSCLC	IIB	0	0
Lung 9	64	Carcinoid Tumor	IA	1	1
Bladder 1	86	TCC	I	1	n/a
Bladder 2	53	SCC	I	1	0
Bladder 3	62	Large Cell Undifferentiated CA	I	1	0
Bladder 4	72	TCC	II	n/a	4
Kidney 1	74	Renal Cell CA	II	3	0
Kidney 2	58	Renal Cell CA	II/IV	2	n/a
Kidney 3	65	Renal Cell CA	III	1	0
Kidney 4	72	Renal Cell CA	III	2	0
Kidney 5	66	Renal Cell CA	II	1	n/a
Kidney 6	52	Renal Cell CA	II/III	2	1
Kidney 7	33	Renal Cell CA	I	n/a	0
Kidney 8	61	Renal Cell CA	II	0	1
Kidney 9	75	Renal Cell CA	II	1	0
Kidney 10	62	Renal Cell CA	I	0	1
Kidney 11	21	Renal Cell CA	II	0	0
Kidney 12	44	Renal Cell CA	I	0	1
Kidney 13	59	Renal Cell CA	II	1	0
Kidney 14	59	Renal Cell CA	I	1 (D-Loop)	0

* all lung body fluids were pre-amplified by WGA

Control samples (blood and matched sputum) were from 12 smokers who did not have cancer. Note that the control population were heavy smokers with a subset (6 of 12) having exposure to asbestos, (Table 2). Patients were considered cancer free based upon spiral CT analysis. DNA in TRIzol^® ^Reagent was recovered using the manufacturer's protocol (Invitrogen Corp., Carlsbad, CA).

**Table 2:** Control cohort: characteristics and number of mtDNA mutations identified

Sample	Age	Pack-years	Asbestos	Blood*/Sputum*
Control 1	53	40	7	0
Control 2	61	72	35	0
Control 3	53	76	33	0
Control 4	43	27	10	0
Control 5	67	40	24	3
Control 6	37	24	0	0
Control 7	59	32.25	0	1
Control 8	49	72	0	0
Control 9	33	29.75	0	0
Control 10	45	52	0	0
Control 11	51	97.5	0	0
Control 12	53	37	4	0

* all control bloods and sputums were pre-amplified by WGA

### STR Genotyping

All samples were genotyped using the PowerPlex^® ^16 System (Promega Corp, Madison, WI) on a 3130xL genetic analyzer with a 36 cm capillary array and POP4 polymer and analyzed using GeneMapper^® ^*ID *v3.2 (Applied Biosystems, Foster City, CA (ABI)). Samples were diluted to 0.5 – 1.0 ng/μL and 1 μL of sample was added to a 24 μL reaction volume (18.2 μL H2O, 2.5 μL 10× buffer, 2.5 μL PowerPlex^® ^16 10× primer pair mix, 0.8 μL (4 U) AmpliTaq Gold^® ^DNA Polymerase (ABI)), then PCR amplified using published conditions. 1 μL of ILS600 internal lane standard and 9 μL of HiDi™ Formamide (ABI) were added to 1 μL of reaction (or 1 μL Allelic Ladder Mix, one for each run) then the mix was briefly denatured and chilled to 95°C and placed on crushed ice for 3 minutes before each sequencing run. Based on STR typing, two tumor isolated mtDNA samples did not match the blood sample from the same patient, and were excluded from the data comparison.

### PCR Amplification- Long PCR

Three primer sets resulting in amplicons of 5–6.1 kb in length were used to amplify the DNA for hybridization onto the Affymetrix Genechip^® ^Human Mitochondrial Resequencing Array 1.0 [50]. Together the primer sets allowed for full coverage of the mitochondrial genome. Due to low DNA quantity recovered from DNA isolation, all 12 matched controls (bloods and sputums from non cancer individials) and 8 BAL samples were pre-amplified by Whole Genome Amplification as described in our previous study [51] prior to long PCR. Amplification was conducted using the TaKaRa *LA Taq*™ Polymerase kit. Briefly, for the MitoChip, the mtDNA template (up to 10 μL), 0.75 μL each of forward and reverse primer (10 μM each), 0.5 μL polymerase (2.5 U), 5 μL LA PCR Buffer with MgCl_2_, 8 μL dNTP mix (2.5 mM each dNTP) and 25 μL of H_2_O were mixed for a total reaction volume of 50 μL. Thermal cycling conditions were as follows: pre-amplification denaturation: (1 cycle), 94°C for 2 min; amplification (30 cycles): 94°C for 15 sec; annealing and elongation, 68°C for 7 min; final elongation (1 cycle), 68°C for 12 min; 4°C hold. None of the bodily fluid samples could be amplified using the three primer pairs, presumably due to quality and/or quantity of mtDNA obtained from these samples. They were successfully amplified using the nine primer pairs previously validated for fluorescent DNA sequencing to provide full sequence coverage of the mitochondrial genome [42]. This required a slight modification in the pooling step of the MitoChip protocol to accommodate nine instead of three amplicons. Each PCR product was visualized on an agarose gel and analyzed for quality and quantity as previously described [17] or by spectrophotometric methods as described in The GeneChip^® ^CustomSeq^® ^Resquencing Array Protocol Version 2.0.

### PCR Cleanup: MitoChip

PCR clean up was conducted using the QIAquick 96 well vacuum plate manifold and protocol [52]. DNAs were eluted in 65 μL of DNAse/RNAse free water.

### MitoChip protocol

The GeneChip^® ^CustomSeq^® ^Resquencing Array Protocol Version 2.0 was used with a few modifications. Briefly, either three or nine amplicons representing the patient and normal control mitochondrial genomes were separately pooled at equi-molar concentrations. Three amplicons were generated from primers provided by JHMI and nine from our previous publication [50]. The PCR amplification products were pooled, fragmented, labeled, hybridized, washed, and scanned. The total quantity of DNA applied to the array was 0.62 μg. Fragmentation of the pooled DNAs was conducted using 0.15 units of Fragmentation reagent (0.033 μL) per sample at 37°C for 15 minutes followed by 95°C for 15 minutes to inactivate. The fragments were labeled with 30 units of TdT at 37°C for 90 minutes followed by 95°C for 15 minutes. The hybridization cocktail, including the separately prepared control fragments, was hybridized for 16 to 18 hours at 45°C rotating at 60 rpm. Arrays were scanned on a GeneArray^® ^2500 Scanner or a GeneChip^® ^Scanner 3000G7 Scanner, and analyzed with GeneChip^® ^DNA analysis (GDAS) and GSEQ softwares.

### MitoChip Sequence Interpretation

Final analysis of all data was conducted using Affymetrix software GCOS v1.4 and GSEQ v4.0. The probe intensities for each mutation reported by the software were examined on the forward and reverse stands for every instance of that base position on the chip. Heteroplamy was identified as 2 bases with probe intensities at least 1.75× the intensity of background for a sequence position. Mutations are defined as instances where the mitochondrial population in the blood for an individual differs from that of the matched sample at a sequence position, such that the base identified in the matched sample is unique and not found as part of the blood mitochondrial population. Mutations (both homoplasmic and heteroplasmic) were confirmed and only reported when the mutation was seen on both strands for locations tiled once on the MitoChip array, and 3 of 4 strands for locations that appear on the chip twice.

### PCR Amplification: NIST Fluorescent Sequencing Protocol

Twelve samples (3 matched specimens from 4 cancer patients) were sequenced by fluorescent sequencing. Amplification was performed in two steps using two different sets of primers (9 matched and 28 matched sets). The second set was needed to obtain amplicons suitable in length for automated fluorescent sequencing. Mitochondrial DNA amplification using the primary (nine primer sets) and the secondary (28 primer sets) nested PCR was previously described [42].

### PCR Cleanup: Fluorescent Sequencing

An enzymatic PCR cleanup protocol was optimized for high-throughput sequencing by enzymatic cleanup using Exonuclease I and Shrimp Alkaline Phosphotase (SAP) (GE Healthcare, Fairfield, CT). To each sample, 1.5 μL of Exonuclease I (1 U/uL) and 1.5 μL of SAP (10 U/uL) was added, then incubated at 37°C for 90 min followed by 15 min at 72°C with a 4°C hold. Samples were subsequently quantified using the Caliper AMS 90 protocol previously described [42].

### Fluorescent DNA Sequencing

The non-coding region (~1000 nt) for each sample was sequenced using CE DNA sequencing, as the MitoChip contains only the coding region. The PCR amplification conditions were as published [42]. PCR amplification primers selected were reported previously to specifically amplify mitochondrial encoded DNA sequences [42,53,54]. Primers contained M13 tags to facilitate DNA sequencing with M13 forward and reverse sequences. Briefly, the blood, tumor, urine, BAL, and sputum mtDNAs were sequenced using the Big Dye™ Terminator (BDT) version 3.1 cycle sequencing kit (ABI). A one eighth cycle sequencing reaction was used for all sequencing. Each reaction contained 1 μL of each of the following reagents: BDT reagent, DNA (3–6 ng/μL), M13 primer (forward or reverse; 5 pmol/μL), 5× Dilution Buffer (ABI), and dH_2_O to a final volume of 5 μL. Cycling sequencing conditions for forward primers were as follows: (40 cycles): 96°C for 10 sec; annealing, 50°C for 5 sec; elongation, 60°C for 4 min; 4°C hold. Reverse primers were sequenced using the same protocol, but the annealing temperature was lowered to 37°C.

The Montage™ SEQ_96 _plate (Millipore Corp., Billerica, MA) was used for cleanup following cycle sequencing. Thirty microliters of Wash Solution (Millipore) was added to each well of the cycle sequencing plate. The samples were transferred to the clean-up plate and placed on the vacuum manifold for 5 to 20 minutes or until the wells were dry. A second wash of 30 μL Wash Solution was added and vacuumed dry for an additional 5 to 30 minutes. Once dry, 20 μL of Injection Solution (Millipore) was added to each well and the plate was mixed vigorously on a plate shaker for 10 minutes.

Resuspended samples were transferred to a 3100 Optical Plate and diluted with 15 μl of HI-DI Formamide (ABI). All separations were performed using the ABI 3130xL Genetic Analyzer using an 80 cm capillary and POP7 polymer system. Samples were electrokinetically injected (30 seconds, 1 kV) and separated at 14.6 kV. Sequences were aligned using the DNA Star SeqMan II (5.05) program and scanned for polymorphisms and sequence variants.

### Statistical Analysis

To determine the statistical significance of mutation and heteroplasmy rates, probability estimates were compared. To test for equivalence the confidence intervals were evaluated for equality at 95% confidence. Distribution of mutations with respect to gene length was tested by Chi square.

## Results

In this study, we evaluated the performance of the Affymetrix Genechip^® ^Human Mitochondrial Resequencing Array 1.0 for detecting mutations present in both tumors and body fluids. Sequence differences between the tumor and the germline (blood) and between body fluid and the germline (blood) were reported as mutations. Sequence differences between the germline and the mtDNA reference sequence tiled on the microarray were considered polymorphisms The microarray software used in this study "learns" as the data set increases, allowing for more statistically significant differentiation between "normal" and "mutant" mitochondrial genotypes [55].

We assessed the DNA mutation spectrum of the entire mitochondrial genome from 72 samples obtained from 26 individuals with different early stage cancers (Table 1). Three matched samples from blood, tumor and body fluid were analyzed from each patient. Six specimens were excluded from comparison due to insufficient sample quantity to perform sequencing (BAL 2, bladder urine 1), insufficient sample to perform the genotyping analysis (kidney urines 2 and 5) or genotypes that could not confirm the specimen to be from the same patient as the blood (bladder tumor 4 and kidney tumor 7); these are designated n/a in Table 1. For the lung cancers, we analyzed mtDNA from primary tumor, brochoalveolar lavage, and blood. For bladder and kidney cancers, we analyzed mtDNA from tumor, urine, and blood. Control samples (blood and matched sputum) were from 12 smokers who were considered cancer free based upon spiral CT analysis. There is overlap in the alveolar macrophages obtained from the sputum and BALs. As reported previously, the long PCR products required for our MitoChip protocol could not be obtained from many of the clinical samples [50]. We therefore used multiple strand displacement amplification to produce sufficient quantities of DNA for application to the arrays. As reported, the amplified material was not altered during multiple strand displacement amplification, retaining the identical heteroplasmic and homoplasmic sequence variants detected in the clinical specimen.

### Mutations detected

MitoChip analysis of the coding region combined with CE analysis of D-loop regions revealed mutations in tumor and body fluid specimens. Tumor samples revealed 18 of 24 speciemens with mutations (6/8 lung [79%]; 3/3 bladder [100%] 9/13 kidney [69%]), for a preliminary sensitivity of 75%. This is significantly higher than observed in prior studies [7]. These data also identified mutations in patient bodily fluids that differed from peripheral blood in 8 of 22 cancer patients; 3/7 BALs, 1/3 bladder urine and 4/12 kidney urine specimens (Table 1). Samples contained one or more mutation. Two of the twelve (17%) sputum specimens from heavy smokers without cancer contained one or more mitochondrial mutations (Table 2); a preliminary specificity of 83%. One specimen contained 3 mutations and the other contained one mutation. Three of these mutations are silent.

A total of 34 mutations were detected in the tumor specimens. Three of the mutations occurred in the D-loop, while the majority of the mutations were spread throughout the coding region. To evaluate whether statistically significant clustering of mutations was found, Chi square values were calculated to compare the location of mutations with the gene targets represented as a proportion of the entire mtDNA genome. For tumor mutations in all genes a nonrandom clustering was suggested for tRNAs and the ND complex genes. The percentages of mutations found within a gene in relation to the percentage of the genome occupied by that gene are; ND complex: 33.4% of the mtgenome and 47.1% of the mutations, tRNA genes: 9.1% of the mtgenome and 17.6% of the mutations, rRNA genes: 15.2% of the mtgenome and 20.6% of the mutations, D-loop: 6.8% of the mtgenome and 8.8% of the mutations, CO: 20.8% of the mtgenome and 2.9% of the mutations, and CytB: 6.9% of the mtgenome and 2.9% of the mutations. There were no mutations detected in the ATPase genes which account for 5.1% of the mtgenome. Thus, mutations more frequently occurred in specific genes, and were statistically rare in the CO genes. Eleven mutations were detected in the body fluids, and the majority of these were heteroplasmic with all mutations occurring in the coding region. The entire list of mutations detected in this study is shown in Table 3, with only purine and pyrimidine heteroplasmies found.

**Table 3:** Cancer cohort mutations detected

		MitoMap Position	Blood	Tumor	Body Fluid	Gene	amino acid/location
Lung	1	1719 G	*	r	*	16S rRNA	-
		2312 A	*	*	r	16S rRNA	-
		3385 A	g	r	g	ND1	Ile>Val
		3450 C	t	y	t	ND1	Pro>Pro
		3480 A	*	r	*	ND1	Lys>Lys
		4901 A	g	r	g	ND2	Gln>Gln
		5773 G	a	r	a	tRNA^Cys^	T arm
	2	3450 C	*	y	n/a	ND1	Pro>Pro
		4580 G	a	r	n/a	ND2	Met>Met
		4901 A	*	r	n/a	ND2	Gln>Gln
	3	16162 A	*	g	*	D-Loop	-
		16284 A	*	g	*	D-Loop	-
	4	10245 T	*	y	*	ND3	Leu(2)>Leu(1)
		13856 T	*	y	*	ND5	Leu_2_>Pro
	5	10427 G	*	r	*	tRNA^Arg^	anticodon arm
		10885 T	*	y	*	ND4	Phe>Phe
		11083 A	*	r	*	ND4	Met>Met
	6	8705 T	*	*	y	ATPase 6	Met>Thr
	7	-	-	-	-	-	-
	9	6413 T	c	c	y	COI	Asp>Asp
		11072 T	*	y	*	ND4	Ser(1)>Pro
Bladder	1	12236 G	*	r	n/a	tRNASer	-
	2	12477 T	*	y	*	ND5	Ser(2)>Ser(2)
	3	1193 T	*	y	*	12S rRNA	-
	4	5169 T	*	n/a	y	ND2	Trp>Arg
		6483 C	*	n/a	y	COI	Leu(2)>Phe
		10668 G	*	n/a	r	ND4L	Ala>Thr
		12714 T	*	n/a	y	ND5	Ile>Ile
Kidney	1	841 A	*	r	*	12S rRNA	-
		2731 T	*	y	*	16S rRNA	-
		12139 T	*	y	*	tRNAHis	acceptor arm
	2	1816 G	*	r	n/a	16S rRNA	-
		3454 G	*	r	n/a	ND1	Ala>Thr
	3	12477 T	*	y	*	ND5	Ser(1)>Ser(2)
	4	841 A	*	r	*	12S rRNA	-
		11394 T	*	y	*	ND4	Leu(1)>Pro
	5	12148 T	*	y	n/a	tRNAHis	D arm
	6	1074 G	*	r	*	12S rRNA	-
		3244 G	*	r	*	tRNALeu	D arm
		14766 C	t	t	*	CytB	Ile>Thr
	7	-	-	n/a	-	-	-
	8	6800 A	*	*	g	COI	Val>Val
	9	15283 T	*	y	*	CytB	Phe>Phe
	10	12471 T	*	*	y	ND5	Ile>Ile
	11	-	-	-	-	-	-
	12	12714 T	*	*	y	ND5	Ile>Ile
	13	7148 T	*	y	*	CO I	Thr>Thr
	14	72 T	*	a	*	D-Loop	-

* : sequence identical to reference sequence

n/a : no data available due to insufficient DNA quantity or STR genotyping results

- : no sequence differences between samples or unknown change to gene

r: purine heteroplasmy (A + G)

y: pyrimidine heteroplasmy (C + T)

### Heteroplasmy

The reported limit of detecting mixed bases or heteroplasmies in solid tumors and body fluids using the MitoChip approaches 2% [50]. We examined the frequency of heteroplasmy in specimens from cancer patients and a control cohort. A total of 202 sequence variants (with respect to the reference sequence) were identified in the mitochondria from the blood of 12 heavy smokers without cancer (Table 4); only 7 of these (3.5%) were heteroplasmic. All seven of these were detected in both blood and sputum and are not haplotype markers. Overall, the rate of heteroplasmy in the control population was quite low; 7 nucleotides in 185,412 nucleotides sequenced, or 0.0038%. The rate of heteroplasmic calls was highest in the tumor DNAs, where 10.5% of all sequence variants detected (43 in 411) were heteroplasmic. In the cancer population, mtDNA heteroplasmies were detected in the blood of 18 of the 26 samples (69.2%) a statistically significantly higher heteroplamy rate than observed in, control blood samples where only 4 of the 12 (33.3%) contained one or more heteroplasmies (Table 4).

**Table 4:** Percent of heteroplasmy calls out of total number of sequence variants.

Genomes sequenced	Number of sequence variants	Number (%) of heteroplsmy sequence variants	Number (%) of specimens with heteroplasmy
Blood from patients with cancer – 26	391	27 (6.9%)*	18 (69.2%)*
Tumor – 24	411	43 (10.5%)*	18 (75.0%)*
BF from patients with cancer – 22	293	11 (3.8%)	7 (31.8%)
Blood from individuals without cancer -12	202	7 (3.5%)	4 (33.3%)
Sputum from individuals without cancer – 12	206	11 (5.3%)	5 (41.7%)

*: Statistically significant difference from non-cancer population and BF

### MitoChip compared with CE DNA Sequencing

The MitoChip sequencing results were compared to bi-directional fluorescent CE DNA sequencing for the 3 matched specimens from 4 patients, a total of 12 samples (Table 5). The MitoChip and CE DNA sequencing results were 99.996% identical (8 differences in 198,816 nt). The percent sequence coverage for the MitoChip was 95.8% (+/-1.4), and the percent sequencing coverage for fluorescent CE sequencing was 97.5% (+/-1.2). Due to MitoChip's increased sensitivity, more mutations were identified by this method than by CE DNA sequencing. All of the 8 differences were heteroplasmic, and 7 of these were detected by the MitoChip and not CE sequencing. The 7 heteroplasmies detected by the MitoChip were in the following gene complexes: 1 CO I, 1 ATPase, 1 ND (all 3: Thr→Ala), 2 tRNA, and 2 silent mutations. The one heteroplasmy detected only by CE DNA sequencing was a silent mutation.

**Table 5:** Nucleotide differences between CE and MitoChip microarray sequencing.

			MitoMap Position	Mitochip	CE	Conflicts
Lung	5	Blood	6050 T	y	*	1
			10427 G	r	*	
		Tumor	10885 T	y	*	3
			11083 A	r	*	
		Bal	6050 T	y	*	1
	7	Blood	10398 A	r	*	2
			12406 G	*	r	
		Tumor	-	-	-	-
		Bal	-	-	-	-
Kidney	3	Blood	-	-	-	-
		Tumor	12477 T	y	*	1
		Urine	-	-	-	-
	14	Blood	-	-	-	-
		Tumor	-	-	-	-
		Urine	-	-	-	

* represents the same base as the reference sequence base

- represents no base differences between samples

## Discussion

Mitochondrial genomes of 26 cancer patients, mostly with early stage (Stage I or II) disease, and 12 heavy smokers without cancer were fully sequenced using the MitoChip and additionally fluorescent CE sequencing for the D-loop region only. DNAs isolated from blood, primary tumor, and BAL for lung cancer patients, from blood, primary tumor, and urine for bladder and kidney cancer patients and blood and sputum from smokers without cancer were sequenced; in total 96 mtDNA sequences were determined. All samples were genotyped then subsequently haplotyped to ensure that samples sets from the same patient were matched (data not shown).

Twenty-three of twenty-six (7/8 lung cancer [88%]; 4/4 bladder cancer [100%] 12/14 kidney cancer [86%]) patients were found to have mtDNA mutations in either the tumor or body fluid or in both, for an overall incidence of 89%. Three of seven (43%) BALs from patients with lung cancer and 2 of 12 (17%) sputums from controls (heavy smokers without cancer) had mtDNA sequence variants, which may result from their heavy smoking [56].

It is interesting to note that the body fluid specimens from the cancer patient population contained fewer mutations overall with respect to the tumors (Table 3). However, it was noted that a heteroplasmic mutation was detected in 3 BALand 3 urine specimens that was not present in the tumor. Additional comparisons between specimens was hampered by the exclusion of sample results due to insufficient sample quality to perform sequencing, insufficient sample to perform the genotyping analysis or genotyping results indicating that the samples were not from the same individual.

The patient cohorts represent a small study and the biological conclusions are therefore provisional pending analysis of larger populations. With regard to incidence of mtDNA mutations in all solid tumors, a greater proportion of lung cancer patients showed a mutation in the tumor compared with kidney and bladder tumor patients. Furthermore, lung cancer patients also showed the highest incidence of mtDNA mutations in bodily fluids (bronchoalveolar lavage), although at a lower incidence than their lung tumors.

An overall summary of the heteroplasmic variation is shown in Table 4. After statistical analysis of these results, it was determined that the tumor samples presented with statistically significantly more heteroplasmies than any of the other populations, including the body fluids from the same cancer patients. Additionally cancer patient blood samples presented with statistically significantly more heteroplasmies than blood from non cancer controls. This suggests the blood samples from these cancer patients do not reflect purely the germline sequence, as heteroplasmic variation has increased in this sample population.

The increase in heteroplasmy is statistically significant, and not considered sample mixup or an artifact in the MitoChip assay. First, as we reported, all samples were initially genotyped then additionally confirmed to be matched after sequencing by mitochondrial haplotyping. Studies conducting MitoChip analysis on manually mixed samples in a dilution series shows the MitoChip assay able to detect sample mixtures without additional false positives in 10% mixtures and down to 2% [43,50]. As the control samples and BALs were pre amplified by Whole Genome Amplificaton (WGA) this must also be considered as a potential source of generating artifacts. In our previous report, we described a MitoChip comparison between pre amplification by (WGA) and genomic DNAs for a subset of the patient cancer and patient non cancer specimens reported in this study. The sequence and mutation profiles for the samples using WGA are reported in this study. In 19 samples compared with and without WGA, there was no significant difference in the sequence obtained; 15 differences were seen in a total of 628,672 tiled positions (19 × 16,544 with and without WGA), and 12 of these samples had no differences [51]. If heteroplasmies resulted from lower specificity of the assay, it is unlikely that identical results would be obtained from separate amplifications (with or without WGA), or be higher in only the cancer patient blood and tumor DNAs.

The origin of observed sequence changes in the mitochondrial genome in cancer remains unclear. Cancer cells develop in a contiguous field of preneoplastic cells that are clonally related to the initial tumor. This study did not detected similar mutations in the body fluids with respect to the tumor. In most samples, at positions of mutation in the tumor, the body fluid sequence matched the mtDNA sequence of the patient's blood sample. This suggests that the body fluid could not sensitively detect the patient's mutations. In 5 patients, heteroplasmic changes were detected in the body fluid that were absent from both patient blood and the tumor samples. The presence of heteroplasmic variance in the body fluid that is absent from the tumor may suggest that systemic oxidative damage maybe occurring.

The D-loop region, which accounts for approximately 6.8% of the genome, contained 8.8% (3 of 34) of the mutations identified in the tumor mtDNAs. Many studies have focused on the short, hypervariable D-loop region, as this region has been reported to have a higher rate of mutation [2]. However, the majority of mutations found in this study were in the coding region, supporting similar findings in more recent studies [6,11]. As reported by Maitra [57], the rationale for preferential sequencing of the non-coding region of mtDNA has been the assumption that this portion of the mitochondrial genome is particularly susceptible to the rigors of DNA damage encountered in metabolically active cancer cells. However, his studies and those of others continue to provide evidence that the coding region is at least equally susceptible to DNA alterations in cancer. In as many as 7 of 14 (50%) preneoplastic samples, somatic alterations restricted to the coding region of mtDNA were detected in that study, while two additional cases had only single non-coding D-loop changes in addition to coding region alterations [57].

The other sequence variants were spread throughout the coding region, with clustering in the tRNA and ND genes (17.6% and 47.1% respectively). The majority of the tRNA mutations were concentrated in the stem region that is under greater selective constraints than loop structures [58]. While an increase in mutations with respect to the ND complex was identified, none of these mutations were found in ND6, a region recently implicated in cancer metastasis [59]. These findings are expected, as the samples in this study were from early stage cancer. Although outside the focus of this investigation, Table 3 shows that certain mutations linked to the ND complex (3450, 4901) were uniquely associated with lung cancer, while other mutations linked to 12srRNA (841) and ND5 (12477, 12714) genes were associated with urologic cancers. This trend is intriguing but it is premature to speculate on implications for tissue specific markers.

We extend the detection of mtDNA mutations as biomarkers of cancer to the detection of mtDNA mutations in body fluids. The combination of a high-throughput sequencing method and non-invasively collected specimens make this potentially an attractive diagnostic amenable to current clinical infrastructure. The usefulness of detecting tumor-specific mtDNA mutations in body fluids requires further investigation for diagnosis and monitoring applications. The use of urine DNA as a source of markers for cancer detection has increasing support [60]. In general, genomic biomarkers that are not tissue specific may be best applied for early detection of recurrence or the detection of potential risk of a secondary tumor site. It is worthwhile to investigate ways to enrich certain cell types, e.g. bladder cells, in order to provide more information than just the presence of cancer. This could be accomplished with antibody capture methods, such as those applied for other cancer biomarkers [61]. While urine analysis cannot indicate the location of a tumor, use of specific washes or fluids opens the vista for diagnostic applications.

## Conclusion

In summary, our results indicate that mtDNA sequencing with the MitoChip can be a high-throughput tool for detecting mutations in clinical samples with potential utility for cancer detection. In this study, 79% of lung,100% of the bladder and 69% of the kidney tumors were found to contain mtDNA mutations; however, these values are based on a limited number of specimens. Our results identified more frequent mutations in the coding region than prior studies with mutation clustering in the tRNA and ND complex genes.

MtDNA mutations may be useful biomarkers, because they affect mitochondrial function, but it is currently unclear the biological relevance of these detected mitochondrial mutations. Our data could not fully substantiate that detection and monitoring of the tumor via mtDNA mutation analysis of body fluids could be a practical way to assess some remote cancer sites. Additional studies are needed to identify the most informative collection procedures and collection site proximity to the tumor. Further, larger scale studies of the mitochondrial genome are needed to compare genotype/phenotype associations in order to understand the pathogenic basis of neoplastic and non-neoplastic diseases linked to mitochondrial dysfunction, and to establish the link between clinico-pathological features and mtDNA mutations.

## Competing interests

The authors declare that they have no competing interests.

## Authors' contributions

JPJ, SM, CDO and PEB performed data analysis and drafted the manuscript. SM, MEM, JPJ, and CDO performed the technical lab work. AKG under WNR and MOH under DS provided the clinical samples. WNR, AM and DS provided clinical expertise. PDW and SS helped with data interpretation and along with PEB provided project leadership. All authors have read and approved this manuscript.

## Pre-publication history

The pre-publication history for this paper can be accessed here:


